# An ingestible bioimpedance sensing device for wireless monitoring of epithelial barriers

**DOI:** 10.1038/s41378-025-00877-8

**Published:** 2025-02-07

**Authors:** Brian M. Holt, Justin M. Stine, Luke A. Beardslee, Hammed Ayansola, Younggeon Jin, Pankaj J. Pasricha, Reza Ghodssi

**Affiliations:** 1 Department of Electrical and Computer Engineering, University of Maryland, College Park, MD 20742 USA; 2 Institute for Systems Research, University of Maryland, College Park, MD 20742 USA; 3 Robert E. Fischell Institute for Biomedical Devices, University of Maryland, College Park, MD 20742 USA; 4 Matrix Lab at A. James Clark School of Engineering, University of Maryland, California, MD 20619 USA; 5 Department of Animal & Avian Sciences, University of Maryland, College Park, MD 20742 USA; 6 Mayo Clinic Hospital, Pheonix, AZ 85054 USA

**Keywords:** Electrical and electronic engineering, Chemistry

## Abstract

Existing gastrointestinal (GI) diagnostic tools are unable to non-invasively monitor mucosal tight junction integrity in vivo beyond the esophagus. In the GI tract, local inflammatory processes induce alterations in tight junction proteins, enhancing paracellular ion permeability. Although transepithelial electrical resistance (TEER) may be used in the laboratory to assess mucosal barrier integrity, there are no existing methodologies for characterizing tight junction dilation in vivo. Addressing this technology gap, intraluminal bioimpedance sensing may be employed as a localized, non-invasive surrogate to TEER electrodes used in cell cultures. Thus far, bioimpedance has only been implemented in esophagogastroduodenoscopy (EGD) due to the need for external electronics connections. In this work, we develop a novel, noise-resilient Bluetooth-enabled ingestible device for the continuous, non-invasive measurement of intestinal mucosal “leakiness.” As a proof-of-concept, we validate wireless impedance readout on excised porcine tissues in motion. Through an animal study, we demonstrate how the device exhibits altered impedance response to tight junction dilation induced on mice colonic tissue through calcium-chelator exposure. Device measurements are validated using standard benchtop methods for assessing mucosal permeability.

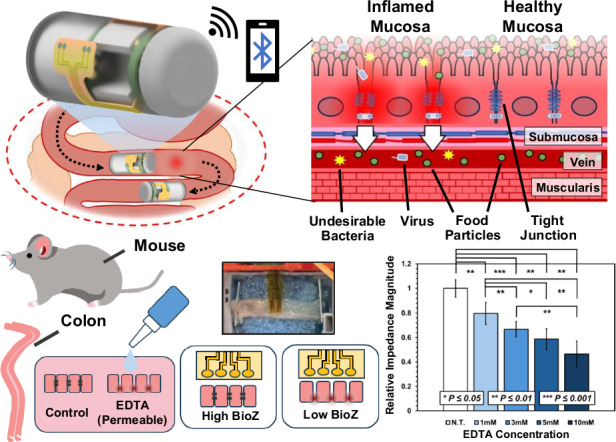

## Introduction

Crohn’s disease (CD) and ulcerative colitis, known collectively as inflammatory bowel disease (IBD), exhibit progressive patterns of inflammation in the gastrointestinal (GI) tract^1^. Immune activation in afflicted intestinal tissue is accompanied by enhanced mucosal permeability, enabling uptake of undesirable bacteria through dilated tight junctions and exacerbating symptoms over time^2^. This condition, deemed “leaky gut,” is implicated in a variety of other diseases such as type 1 diabetes, celiac disease, multiple sclerosis, and autoimmune disorders^3^. Though the physiology of leaky mucosal tissue is well-studied, existing devices are unable to explicitly characterize the local onset and progression of mucosal barrier dysfunction beyond the esophagus. In a large-scale study on 1420 healthy relatives of CD patients, elevated intestinal mucosal permeability predicted onset of the disease by year^4^ Endoscopy is extensively used for visualization of gastrointestinal tissues in IBD patients, guiding therapy and detection of dysplasia (i.e., precancerous lesions)^5^. However, endoscopes lack access to long sections of the GI tract^6^ and cannot be used to measure gut leakiness or subclinical inflammation, warranting development of innovative sensing platforms to facilitate local measurement of mucosal barrier integrity. Currently, quantification of mucosal permeability is achieved using transepithelial resistance (TEER) via cell culture^7^ or Ussing chamber experimentation with excised tissues^8^. Yet, these methods cannot be used clinically without the need for biopsy or invasive mucosal sampling. Intestinal permeability may be non-invasively measured in patients through molecular probes, such as lactulose-mannitol and sucrose, through ingestion and analysis of excreted urine samples^9^. Still, this technique is non-specific and unable to locally identify patterns of afflicted epithelial tissues in vivo. Hence, tools for localized monitoring of tissue properties linked to epithelial permeability remain in high demand.

Ingestible devices have emerged as a burgeoning alternative to endoscopy or esophageal catheters, enabling continuous visualization or measurement in inaccessible regions of the GI tract^10^. Ultrasound^11^ or video endoscopy capsules^12^, such as the PillCam™, have been developed for visualizing tissue and are widely used clinically to support disease diagnosis. However, visualization alone is often not sufficient for locally evaluating tight junction integrity and quantifying disease progression. Toward this end, physiological sensing capsules have been proposed for measuring biomarkers such as pH^13^, intestinal gases^14^, temperature^15^, and motility^16^, yielding the benefit of continuously reporting wireless numerical values for diagnostics or real-time feedback^17^. Still, many of these sensing modalities cannot provide explicit tissue properties applicable toward mucosal barrier characterization. Bioimpedance sensing, analogous to TEER, can be used for measuring tissue conductivity and characterizing paracellular ion permeability^18^. In inflamed intestinal mucosal tissue, changes in tight junction protein content widen the paracellular pathway^19^, enhancing ion permeability and reducing tissue impedance^20^. According to Gitter et al., mucosal conductivity has been observed to increase above baseline in proportion to the severity of inflammation and permeability in Ulcerative Colitis patients, with increases of 39.3 ± 7.1% in mild cases and 409.5 ± 71.8% in severe cases of colonic inflammation. In established methods such as TEER and Ussing chamber measurement, electrodes are placed on opposing sides of tissue to transmurally generate an excitation electric field^21^. In a non-invasive manner, coplanar bioimpedance electrodes for current injection and voltage sensing may be employed within the lumen to assess mucosal conductivity across a spectrum of frequencies, isolating extra- and intra-cellular resistivity and phase^22,23^. Paracellular permeability, linked to leaky gut, is defined by the ion or molecule flow through cryptic tight junctions^24^. In electric cell-substrate impedance sensing (ECIS), excitation frequencies above 10 kHz prioritize transcellular ion transportation, and the paracellular pathway is primarily monitored below 400 Hz^25^. In Wegener et al., the 200 Hz – 5 kHz interrogation frequency range was identified as optimal for monitoring tight junction formation, as lower frequencies were dominated by interfacial noises and higher frequencies were not adequately sensitive^26^. Although these measurement frequencies are applicable to ECIS, sensor contact varies with wearable and ingestible bioimpedance probes motivating a wider range of potential measurement frequencies for optimizing sensitivity. For example, a fixed frequency of 9.6 kHz has previously proven effective at differentiating inflamed mucosal tissue in patients with Barrett’s Esophagus^27^. Generally, low excitation frequencies ( < 10 kHz) prioritize paracellular pathways; however, this has not yet been verified in an ingestible capsule context. In this work, we investigate the optimal frequency for measuring induced tight junction damage across fifty frequencies spanning 100 Hz – 200 kHz.

Despite the wide array of ingestible technologies, few explore GI tissue impedance, and no existing devices can link tight junction dilation to real-time changes in tissue impedance beyond the esophagus. Previous bioimpedance sensing ingestible devices have been proposed towards detection of gastroesophageal reflux disease (GERD)^28^ and eosinophilic esophagitis (EoE)^29^. For example, Goffredo et al. developed an ingestible device for general-purpose closed-loop drug delivery utilizing ring-like impedance electrodes for differentiating healthy and pathological tissues^30^. Recently, Bettinger et al. demonstrated a tethered bioimpedance sensing capsule capable of differentiating healthy and damaged esophageal mucosa via external potentiostat electronics^31^. However, these systems perform two-probe impedance measurements using polarizable electrode materials, introducing measurement noise at low excitation frequencies due to interfacial^32^ and electrode-electrolyte impedances^33^ at low interrogation frequencies. Previous studies comparing gastrointestinal tissue impedance based on permeability or tissue inflammatory state have also been conducted in the esophagus^34–36^; but comparable datasets from the small or large intestine are lacking. Hence, few non-invasive tools exist for monitoring GI mucosal barrier integrity using bioimpedance.

There are several challenges that have limited the applicability of bioimpedance as a sensing modality in ingestible devices, including sensor integration with low-power electronics, measurement noise caused by interfacial impedances, and challenging GI environmental conditions. While sensor configuration may be tailored for targeted epithelial monitoring using compact electrodes, measurement fidelity suffers using low-power potentiostat electronics due to high interfacial impedances inherent in microelectrodes^37^ comprising polarizable metals. Some of these materials, including Au and Pt, are highly biocompatible and electrically conductive; however, their implementation in bioimpedance sensing obscures physiological information at low excitation frequencies due to limited charge transfer capability (CTC). For sensitive measurement, sufficient electric charge must be transferred from the metallic electrode to aqueous ions in tissue through capacitive charge injection across the electrical double-layer or Faradaic redox reactions^38^. Unmodified metallic electrodes often cannot overcome electrode-electrolyte impedances^39,40^. Surface treatments, including Pt black^41^, carbon nanotubes^42^, or conductive polymers such as polyaniline^43^, polypyrrole^44^, or poly (3,4-ethylenedioxthiophene) with polystyrene sulfonate dopant (PEDOT:PSS)^45^ are extensively used in bioelectronic applications due to minimize these interfacial impedances and enhance CTC, reducing noise^46^. In the distal intestine, measurement variability is highly undesirable due to shifting electrode position and capsule orientation during peristalsis^47^. Hence, effective impedance sensing for monitoring barrier integrity in the GI tract demands targeted sensor design and surface treatment, along with integration with miniaturized potentiated circuity capable of wireless communication in a compact form factor.

**Fig. 1:**
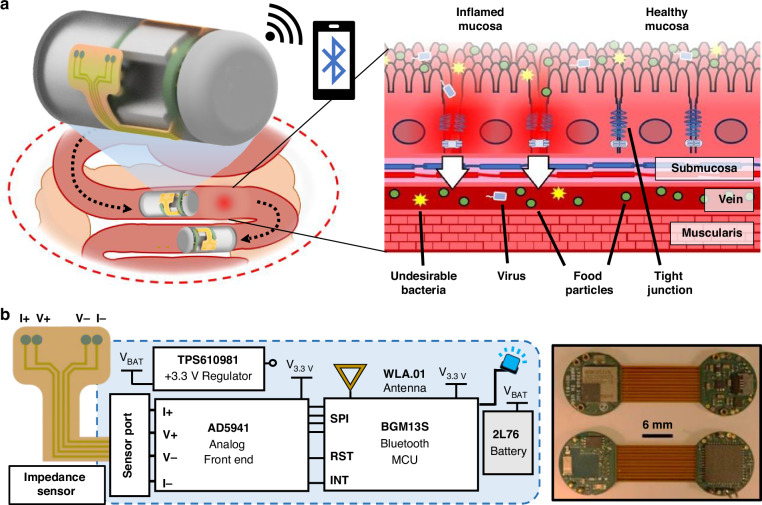
Overview schematic depicting the ingestible capsule monitoring bioimpedance in the small intestine. **a** Diagram of an ingestible bioimpedance sensing device traversing through the GI tract, wirelessly transmitting impedance. Illustration of tight junction dilation during inflammatory processes in the small intestinal mucosa. **b** Electronics schematic of impedance sensing capsule and flex-rigid PCB

In this work, we address these challenges and demonstrate a noise-resilient bioimpedance sensor coated with a PEDOT:PSS film for integration with ingestible capsule platforms, enabling characterization of mucosal ion permeability continuously throughout the GI tract (Fig. 1a). We explore various PEDOT:PSS treatments by altering applied current density and validate the performance of the coated sensors in saline and on excised porcine intestinal tissue. The integrated capsule device successfully differentiated tissue soaked in 1x phosphate-buffered saline (PBS) from untreated tissue using the coated sensor, reporting significant reduction in impedance magnitude and reducing measurement variation obtained with the uncoated sensor. During continuous translation across porcine tissue, the device wirelessly reports impedance magnitude, activating an LED in response to reduction below a wirelessly programmed magnitude threshold. Finally, through testing on a small animal trial consisting of excised mice colonic tissue, we validate that the sensor can differentiate healthy and permeable tissues at benchtop and following integration with the capsule device. This work signifies major progress towards the use of non-invasive bioimpedance sensing as a diagnostic tool in ingestible technology and leaky gut identification.

## Results and discussion

### Electronic circuit design and capsule operation

For impedance sensing via an ingestible device, circuitry is needed to generate and record an electrochemical impedance spectroscopy (EIS) waveform and wirelessly transmit data with minimal power consumption. As shown in Fig. 1b, the device electronics were fabricated onto a custom flex-rigid printed circuit board (PCB) described in our prior work^48^. The system features an electrochemical analog front end (AFE), AD5941 (Analog Devices, Wilmington, MA), to generate the excitation signal and perform four-wire impedance measurements. It also includes a Bluetooth Low Energy microcontroller (BLE-MCU), BGM13S (Silicon Labs, Austin, TX), and integrated 2.45 GHz chip antenna, WLA.01 (Taoglas, San Diego, CA), for data processing and wireless communication. Data is received through the EFR Connect phone app (Silicon Lab, Austin, TX) twice per second. This enables wireless control of impedance measurement parameters such as interrogation frequency during transit through the GI tract. Additionally, a magnetic reed switch is connected between the battery and PCB to conserve power when not in use. Lithium-based and silver-oxide (Ag_2_O) batteries are commonly used energy sources for medical implantable and ingestible devices and are safe when connected to battery protection circuitry and securely packaged^49^. Though Ag_2_O batteries have been already approved for clinical use in video capsule endoscopy, a lithium-manganese dioxide (LiMnO_2_) battery was chosen for its high capacity and compact size, spanning 11.6 mm in diameter and 10.8 mm in height. The battery is capable of continuously powering onboard electronics for 29 h (Supplementary Table 1). The battery, reed switch, and PCB are housed within a 3D-printed capsule shell measuring 13.8 mm in diameter and 27.9 mm in length, aligning with previously developed ingestible devices evaluated in porcine models (Supplementary Table 2). A four-pin flat-flex cable (FFC) port is included on the side of the PCB along with a rectangular intrusion in the shell to interface the fabricated sensor with the AD5941. Finally, a rounded cap was added, and the assembly is sealed with hot-melt adhesive for testing.

The AD5941 potentiostat integrated circuit (IC) can perform electrochemical impedance spectroscopy (EIS) across a frequency range between 100 Hz and 200 kHz boasting measurement error within 2.8% of a standard benchtop impedance analyzer^50^. The developed capsule measures impedance magnitude at a single excitation frequency than can be wirelessly controlled to maximize the sampling rate during transit. EIS measurements are initially performed during benchtop experimentation with the wired potentiostat evaluation board (EVAL-AD5941) and subsequently verified using the developed PCB and assembled capsule. The EVAL-AD5941 communicates data from the sensor to a PC using the SensorPal GUI (Analog Devices, Wilmington, TX) software. For single-frequency measurements the optimized frequency range was determined to be between 10 kHz and 100 kHz, as measurement efficacy suffered below 1 kHz and above 100 kHz on solid media using the EVAL-AD5941 (Supplementary Fig. 1). The AD5941 potentiostat supports tetrapolar bioimpedance measurements, reducing signal noise by separating the contributions of current injecting and voltage sensing electrode pairs. However, at very low frequencies, measurements are susceptible to noise fluctuations based on applied contact pressure^51^ and electrode-electrolyte capacitances^52^ attributed to polarizable metal electrodes. These interferent conditions may be resolved through electrode surface modification and by utilizing the frequency-sweep feature of the AD5941 to identify an optimal measurement range. Additionally, a notable challenge for ingestible devices is wireless signal attenuation through GI tissues, primarily abdominal muscle, hindering data acquisition once ingested. Communication using BLE protocols has been previously verified in a worst-case tissue analog^48^. In this study, the device was surrounded by 10 cm of 88% lean beef on all sides, demonstrating adequate signal strength (> −100 dBm RSSI) up to 60 cm beyond the analog surface, making it suitable for transmission to nearby mobile devices or bedside recording equipment.

### Design of PEDOT:PSS-coated bioimpedance sensor

**Fig. 2:**
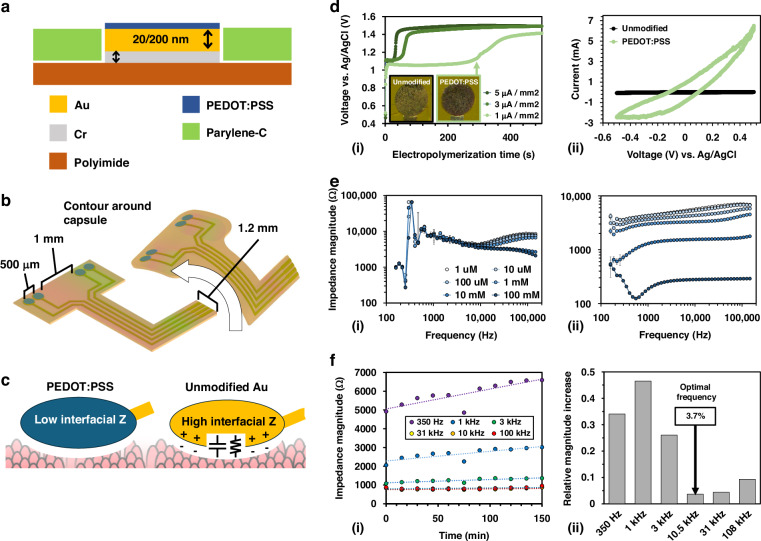
Sensor illustration and PEDOT:PSS electrodeposition. **a** Schematic diagram of cross-sectional view of sensor. **b** Exploded rendering of impedance sensor bending to conform to cylindrical package. **c** Illustration of PEDOT:PSS treatment on sensor, reducing interfacial impedances during impedance measurement on epithelial tissue. **d** (i) Chronopotentiogram of PEDOT:PSS electrodeposition onto Au electrodes at various current densities. (ii) Cyclic voltammogram of unmodified and PEDOT:PSS-coated Au electrodes (1 µA·mm^−2^, 600 s) in PBS (Scan rate: 100 mV·s^−1^). **e** EIS recording via EVAL-AD5941 portable potentiostat development kit of (i) unmodified and (ii) coated sensor (*N* = 3), exhibiting uniform frequency response and excellent sensitivity to ion content. **f** PEDOT:PSS-coated sensor performance after repeated EIS measurements in PBS: (i) impedance magnitude response to time at various frequencies, and (ii) relative increase in impedance magnitude from initial measurement after 150 min

A four-probe Wenner-Schlumberger electrode array is used to greatly reduce current-injecting electrode impedance contributions and maximize tissue sensitivity by separating injecting and voltage-sense electrode pairs^53^. In Ussing chamber measurement, the electric field for probing passes transmurally across tissue, enhancing the impedance measurement contribution of non-epithelial tissue layers. Using coplanar electrodes, the sensor geometry parameters including electrode size and spacing may be optimized to confine the excitation field and prioritize mucosal impedance contributions. Compact electrode geometries are ideal for enhancing measurement sensitivity at limited tissue depths but compound interfacial impedances^37^. A finite-element method (FEM) model was devised to assess current density and electric field propagation through stacked intestinal tissue layers with dielectric properties obtained from previous literature^54^. In the model, sensor geometry was optimized to selectively report impedance alterations in the mucosal tissue layer using an electrode width, *W*, of 500 µm and voltage-sensing electrode distance, *D*, of 1 mm (Supplementary Fig. 2). Various sensor geometries, with electrode sizes ranging between 500 µm and 2 mm and spacing ranging from 2 mm to 8 mm, were fabricated using lift-off (Supplementary Fig. 3). EIS was performed using each sensor in potassium chloride (KCl) with varying ion concentration. Ionic agarose tissue phantoms were devised with varying thickness and ion content and compared to verify depth-targeting capability of compact sensors (Supplementary Fig. 4). Wide (4–8 mm) inner spacing between voltage-sense electrodes resulted in higher impedance magnitude, confirming that sensor width modifies the volume of targeted tissue. Large (1– 2 mm) sensor electrodes yielded significantly lower impedance values due to reduced interfacial impedances. Conversely, smaller electrodes (500 µm) with compact inner spacing ( ≤ 4 mm) exhibited higher impedance magnitude but minimal variation, confirming diminished impedance contributions beyond 500 µm of ionic target media. Figure 2a shows the schematic diagram of the bioimpedance sensor, composed of a chromium (Cr, 20 nm) adhesion layer and gold (Au, 200 nm) metallic electrode, patterned onto a flexible polyimide substrate for bending around the capsule packaging (Fig. 2b). Benchtop testing on agarose phantoms was conducted using a Gamry Interface 1010E potentiostat (Warminster, PA). A biocompatible polymer, Parylene C (1.2 µm thickness), is used to insulate the signal traces from the surrounding environment. The exposed electrodes are coated in a highly stable conductive polymer, PEDOT:PSS, using electrodeposition^55^ (Supplementary Fig. 5) to resolve high interfacial impedances (Fig. 2c). Chronopotentiometry recordings, shown in Fig. 2d (i), depict the electrode voltage response, relating applied current density (1, 2, and 5 µA·mm^−2^) to the formation of the PEDOT:PSS film. During electrodeposition, the sharp increase and subsequent plateau of the electrode voltage was attributed to the formation of the polymer film. A slight discoloration of the electrodes was observed following treatment (Fig. 2d (i) inset), confirming successful film formation^56^. The CTC of PEDOT:PSS-coated electrodes were characterized using cyclic voltammetry (CV) in a 10 mL solution of 1x-Phosphate Buffered Saline (PBS) using a benchtop potentiostat. Voltage was swept from −0.5 V to +0.5 V using a standard Ag/AgCl reference and Pt counter electrode configuration using three cycles. Figure 2d (ii) shows the cyclic voltammograms of PEDOT:PSS-coated electrodes, using an applied current density of 1 µA·mm^−2^, compared to unmodified electrodes. This resulted in a 51.4-fold increase in current response, reflecting enhanced CTC for excitation field generation through tissue^45^. Electrodes were simultaneously subjected to a fixed current density of 1 µA·mm^−2^ for electrodeposition to achieve a reliable PEDOT:PSS film (Supplementary Fig. 6). Additionally, the PEDOT:PSS working cell was replaced between sensors to ensure effective treatment. Re-use of the working cell yielded diminished CTC with longer electrodeposition time (Supplementary Fig. 7).

### Characterization of PEDOT:PSS-coated bioimpedance sensor in solution

High interfacial impedances contribute to measurement variability and obscure the relationship between ion permeability and observed impedance. Experimentation was initially performed to assess ion sensitivity in aqueous media to quantify noise resolution achieved through PEDOT:PSS treatment. Using the EVAL-AD5941, a full-frequency EIS sweep was performed to characterize the impedance magnitude response of untreated (Fig. 2e (i)) and PEDOT:PSS-coated (Fig. 2e (ii)) sensor in KCl solutions. The PEDOT:PSS-coated sensors exhibited distinguishable impedance values, uniform frequency response, and sensitivity to sub-10 µM ion concentrations, ideal for application in vivo. For the uncoated sensor, measured impedance magnitude was indistinguishable at frequencies below 10 kHz when subjected to varied ionic solutions as shown in Fig. 2e (i). At high frequencies, the magnitude difference between 1–10 mM KCl solutions was inconsistent with other concentrations. Since stable measurements were not achieved below 2 kHz for either sensor, this frequency range was not considered for fixed-frequency measurement via the ingestible device.

Repeated measurement and exposure to conductive media may affect the integrity of the PEDOT:PSS film over time. PEDOT:PSS lacks long-term chemical stability in ionic conditions, as PSS is a hydrophilic counter-anion leading to PEDOT moisture-absorption and de-doping of the film over time^57^. Impedance spectroscopy at an excitation frequency of 1 kHz has been reported as a good metric for quantifying electrochemical electrode properties^58^. EIS was performed using the PEDOT:PSS-coated sensor at select frequencies including 1 kHz in 1x-PBS to evaluate how impedance magnitude changes with time in aqueous media. In Fig. 2f (i), the magnitude response exhibits drift at lower frequencies but shows excellent stability over time at 10 kHz and beyond. The magnitude slightly increased over time, confirming de-doping of the PEDOT:PSS film as denoted by higher interfacial impedance. At low frequencies below 3 kHz, the sensor experienced a relative impedance magnitude increase between 26.1–46.5% during measurement, whereas higher frequencies experienced less than 10% time-dependent variation. An interrogation frequency of 10.5 kHz was empirically determined to minimize relative alterations in impedance as shown in Fig. 2f (ii). At this frequency, impedance magnitude only increased 3.7% from its original value. Ultimately, the observed time-dependent increase in impedance magnitude using the fabricated sensor and EVAL-AD5941 is acceptable for prolonged measurement in ionic conditions. Even minimal tight junction dilation was found to reduce impedance by 20.3 ± 9.0% on average, exceeding time-dependent impedance variation at the target frequency. Future investigation must be conducted to quantify changes in CTC of the PEDOT:PSS film during capsule traversal through various GI regions. Despite the effect of pH on of PEDOT:PSS film conductivity^59^, previous studies employing PEDOT:PSS for electrical measurement on various gastric tissues have reported excellent stability^60,61^. In the future, CV and EIS measurement in simulated intestinal fluid for prolonged periods may be used to elucidate stability of the PEDOT:PSS film in realistic GI conditions. Alternatively, to reduce pH-dependent effects on PEDOT:PSS film conductivity^62^, stomach acid inhibition may be achieved through proton pump inhibitor pre-treatment in patients.

### Ex vivo validation of capsule device

**Fig. 3:**
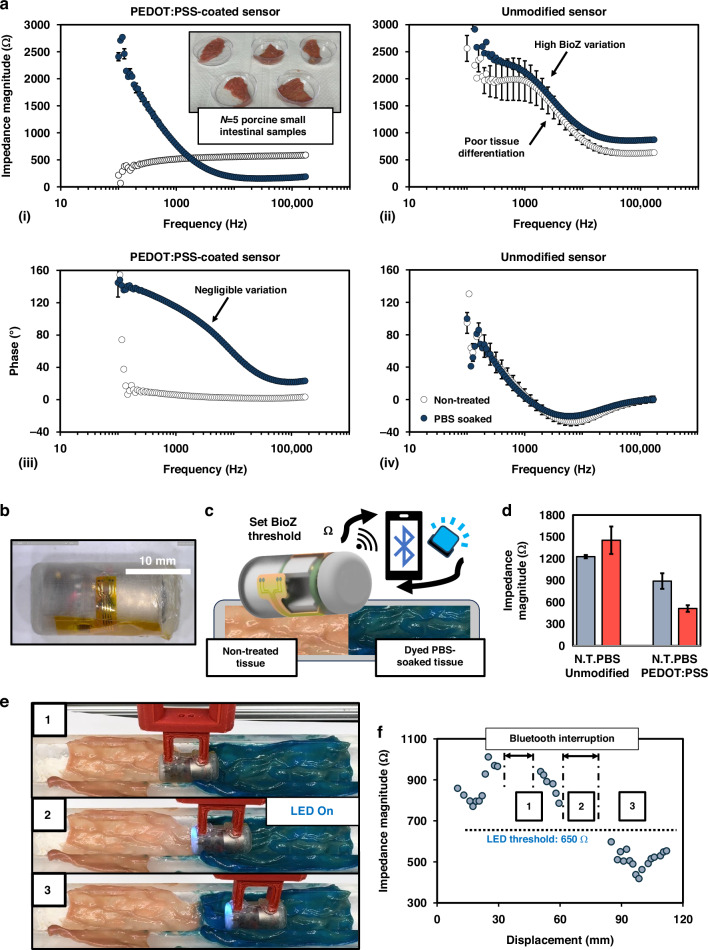
Comparison of untreated and PEDOT:PSS-coated electrodes on excised porcine intestinal tissue samples. **a** EIS impedance magnitude recordings were obtained using the AD5941 devkit on five porcine small intestinal tissue samples with (i) PEDOT:PSS-coated and (ii) unmodified impedance sensors. The phase response was measured for (iii) PEDOT:PSS-coated and (iv) unmodified impedance sensor. **b** Photograph of assembled sensor-integrated capsule. **c** Illustration of capsule device obtaining initial measurements to wirelessly set bioimpedance LED threshold. **d** Average impedance magnitude reported for unmodified tissue and PBS-soaked tissue by Au and PEDOT:PSS-coated sensor following capsule-integration. **e** Linear translation of integrated capsule measuring impedance along porcine small intestinal tissue (pink) and dyed PBS-treated tissue (blue) depicting LED activation upon contact with treated tissue. **f** Impedance magnitude data transmitted to mobile device via Bluetooth during translation

PEDOT:PSS treatment significantly improved the ion sensitivity of unmodified Au in aqueous media. Prior to integration with capsule electronics, the ability of the PEDOT:PSS-coated sensor to identify ion-permeable intestinal tissues must first be verified using the EVAL-AD5941. EIS was performed between 100 Hz and 200 kHz on excised, commercially obtained porcine small intestinal tissue (*N* = 10) using the EVAL-AD5941 to assess measurement variability. A subset of samples (*N* = 5) were soaked in 1x PBS for 24 h to enhance tissue conductivity, representing ion-permeable tissue. Figure 3a shows the full-spectrum impedance response of an unmodified and PEDOT:PSS-coated sensor. The unmodified sensor exhibited an indistinguishable impedance response between untreated and PBS-soaked tissues. Conversely, the PEDOT:PSS-coated sensors yielded a significant magnitude difference between each group and boasted a reduction in sample-to-sample variation. This behavior is expected, as standard benchtop experimentation has previously demonstrated that PEDOT:PSS treatment reduces measurement variation experienced by bare gold electrodes^63^. Sampling at 10 kHz, the PEDOT:PSS-coated sensor generated considerably lower impedance magnitude values for both groups and decreased magnitude variation 58.7% and 68.3% on control and treated porcine small intestinal tissue.

Following benchtop testing of the PEDOT:PSS-coated sensor on intestinal tissue, the capsule device was assembled, as shown in Fig. 3b. The system was initially validated using an unmodified Au sensor (Supplementary Fig. 8). Here, impedance magnitude proved sensitive to orientation, limiting its efficacy during GI transit. Noise resolution through PEDOT:PSS treatment was then evaluated through capsule translation across tissue, comprising unmodified and dyed PBS-treated porcine small intestinal tissues pinned to a silicone backing (Ø = 25 mm) mimicking the intestinal lumen. A custom linear actuator was constructed to control the speed, contact pressure, and position of the capsule in relation to the pinned tissue, simulating peristaltic motion in the GI tract^64^ (Supplementary Fig. 9). While the linear actuator system is limited to linear motion and cannot explore shifting capsule orientation or flexures, given the relative diameter of the human small intestine and capsule dimensions, sensor contact is expected to be maintained during peristaltic contractions. Inconsistent contact with the intestinal lining or introduction of bolus across the electrodes would result in spurious fluctuations in the bioimpedance magnitude, or noisy data, potentially making quantification of tissue permeability difficult.

Wireless bioimpedance magnitude was recorded while moving across the tissue track using an excitation frequency of 10 kHz with a sample rate of 2 Hz. The BLE-MCU was programmed to activate an onboard LED in response to a reduction in impedance magnitude below a threshold value to confirm that the device can differentiate each tissue segment. This value was wirelessly set via a Bluetooth command as shown in Fig. 3c. Based on initial measurements with the device, the LED threshold was set to 650 Ω (Supplementary Fig. 10). Once the command was received, the device dynamically activated the blue LED when placed on PBS-soaked tissue and deactivated during contact with untreated tissue (Supplementary Video I). Sampling at 10 kHz, the reported impedance magnitude was invariant to contact pressure on both tissue groups using the PEDOT:PSS-coated sensor. Wireless measurements received through the EFR Connect app confirm the device’s ability to successfully differentiate tissue state during linear translation using the PEDOT:PSS-coated sensor. Average impedance values collected using the assembled capsule device on each tissue group using the untreated and treated sensors during translation are compared in Fig. 3d. A significant reduction in bioimpedance magnitude (N.T.: 890 ± 107 Ω, PBS: 513 ± 45 Ω) was observed with low variation during translation. However, capsule translation across the tissue using the unmodified Au sensor resulted in a near identical impedance magnitude response (N.T.: 1227 ± 23 Ω, PBS: 1453 ± 189 Ω), mirroring stationary impedance measurements obtained using the EVAL-AD5941. Figure 3e depicts visual confirmation of the capsule as it transitions from (1) the non-treated (N.T.) tissue, (2) initially contacts the dyed PBS-soaked tissue, and (3) continues traversal. LED activation upon contacting PBS-soaked tissue demonstrates that the BLE-MCU received the wirelessly programmed bioimpedance activation threshold, showing that the device is compatible for feedback-driven applications. Brief interruptions in Bluetooth transmission prevented impedance magnitude from updating during two intervals (Fig. 3f). Since no new values were reported, this data was extracted following the measurement and the interruption had no effect on LED activation (Supplementary Video II). This result confirms that stable impedance magnitude values can be wirelessly achieved through four-probe sensing using the PEDOT:PSS-coated sensor and capsule electronics. As expected, the unmodified Au sensor was incompatible for integration with the capsule device, as no significant difference was observed during translation across saline-soaked tissue. Future work will address the challenge of inconsistent sensor contact by developing motor-driven actuators to synchronize repeated capsule anchoring with data acquisition, greatly enhancing the reliability of the sensor measurement and reducing motion-induced measurement variation. Such modification will enable consistent applied pressure on the sensor, improve contact by displacing luminal content obstructing the mucosal wall, and offer higher resolution by slowing capsule transit.

### Bioimpedance response of tight junction damage in mucosal tissue

**Fig. 4:**
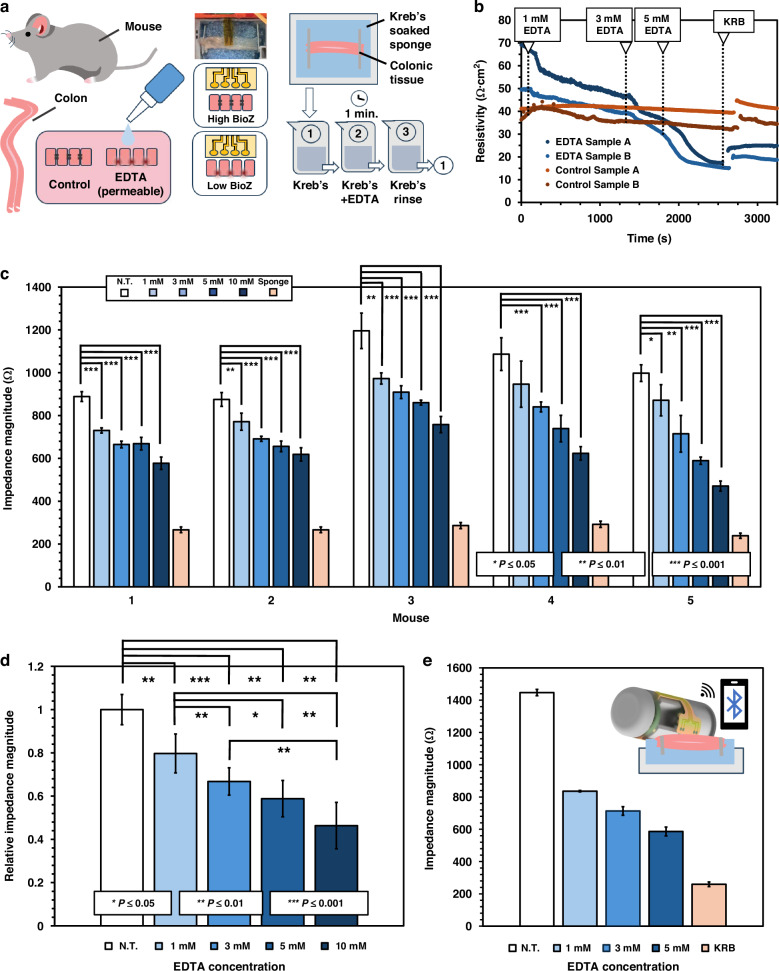
Mice experimentation overview in Ussing chamber and using fabricated sensor with and without integration with capsule electronics. **a** Mice colonic tissue is extracted and treated with EDTA to disrupt tight junctions, reducing transepithelial resistance and hence mucosal bioimpedance. **b** Ussing chamber measurement confirms varying degrees of decreased tissue resistance following EDTA treatment over time for EDTA-treated samples A and B, and constant tissue resistance for the two control samples. When EDTA is replaced with KRB solution, tissue resistance slightly rises, denoting recovery. **c** Bioimpedance magnitude values sampled at 10.5 kHz among five mice using EVAL-AD5941. **d** Averaged magnitude values plotted relative to healthy, non-treated tissue impedance **e** Assembled capsule measurement at 10 kHz

At low excitation frequencies, impedance spectroscopy on tissue probes the extracellular space^65^ which may be used to target tight junction dilation and enhanced paracellular conductivity during inflammatory processes. An animal study is performed on mice colonic tissue ex vivo to verify that the sensor and capsule device can characterize changes in impedance caused by opening of tight junctions. Previous studies have investigated acid-induced esophageal damage, compromising the integrity of mucosal cells^31^. An animal study was devised to isolate tight junction damage in large intestinal tissue, representative of IBD. Here, tight junction dilation is induced through the application of ethylenediaminetetraacetic acid (EDTA), a calcium chelator that enhances mucosal paracellular permeability (Fig. 4a)^66^. The degree to which EDTA affects mucosal permeability is highly dependent on dosage concentration^67^ and exposure duration^68^. TEER was measured on a subset of samples using an Ussing chamber (Fig. 4b) to confirm that the EDTA challenge manifests mucosal impedance alterations. Colonic samples (*N* = 4) were placed into the chamber and preserved in oxygenated Kreb’s-Ringer solution. EDTA was applied in increasing concentration to both hemichambers for two samples denoted EDTA-A and EDTA-B, and the other two tissue samples Control-A and Control-B remained unchanged. Figure 4b shows the TEER measurement of all four samples, confirming enhanced paracellular permeability from EDTA exposure. Increasing EDTA concentration results in sharper reduction of mucosal impedance until a plateau is observed at approximately one-third of its initial value. Finally, for all samples, the fluid is removed and replaced with fresh KRB, resulting in recovery and higher TEER for EDTA-A and EDTA-B.

EIS measurements are performed using the EVAL-AD5941 on colonic tissue extracted from five mice before and after treatment with 1, 3, 5, and 10 mM EDTA for one-minute intervals (Supplementary Fig. 11). First, experimentation was performed to verify that EDTA alone has no effect on measured impedance (Supplementary Fig. 12). Tissue is pinned to a porous biopsy sponge soaked in Kreb’s-Ringer Buffer (KRB) for preservation. Measurements were performed at frequencies between 100 Hz to 200 kHz. At an interrogation frequency of 10 kHz, impedance magnitude consistently decreases from applied EDTA. Measurements below 1 kHz were dominated by interfacial impedances, and frequencies above 50 kHz did not consistently reflect incremental tight junction widening for all mice. While lower frequencies (100 Hz – 1 kHz) are best for mucosal barrier characterization in ECIS, a slightly higher frequency range is warranted for ingestible devices due to variable contact. This aligns with common single-frequency bioimpedance analysis (SF-BIA) practices, where an interrogation frequency of 50 kHz is standard^69^. During measurement, sensor position and contact pressure are kept constant through application of a 2 g weight during measurement. Sampling at an optimal frequency of at 10 kHz, data are represented relative to baseline impedance magnitude values of healthy tissue as shown in Fig. 4c to account for variation in tissue thickness and basal impedance. The average impedance of the biopsy sponges remained consistent among various sensors (270.0 ± 18.7 Ω), confirming that measurement variation could be attributed to physiological factors. EIS measurement using fabricated sensors denotes similar behavior to Ussing chamber results at sampling frequencies above 1 kHz, as increasing EDTA concentration corresponded to incremental decreases in impedance magnitude in all mice. At an interrogation frequency of 10 kHz, the KRB-soaked sponge impedance was deducted for all mice and relative impedance values averaged among all five mice are plotted in Fig. 4d. Impedance magnitude was reduced to 79.7 ± 9.0, 66.8 ± 6.3, 58.8 ± 8.4, and 46.4 ± 10.7% of its original value under four EDTA challenges (1–10 mM). The observed behavior confirms that the developed sensor is capable of reporting impedance alterations in conjunction with mucosal tight junction dilation with sufficient sensitivity to detect mild cases of colonic inflammation^20^.

Finally, the impedance magnitude of excised colonic tissue obtained from a separate mouse was wirelessly reported using the assembled device for comparison with trends observed through Ussing chamber and EVAL-AD5941 measurement. A similar EDTA challenge was applied to tissue at three concentrations ranging from 1–5 mM. Figure 4e presents the average values obtained from wireless measurements using the assembled capsule device when placed onto the tissue sample. The assembled device reported a decrease in bioimpedance of 42.2 ± 0.3, 50.7 ± 1.8, and 59.5 ± 1.9% in response to 1-, 3-, and 5 mM concentrations of EDTA, respectively, confirming that the device can differentiate leaky and healthy colonic tissue. The wirelessly measured response in KRB solution is 259.3 ± 14.4 Ω, closely aligning with values obtained using the EVAL-AD5941. Similarly, average impedance magnitude values reported from the capsule for 1, 3, 5 mM treatment (836.0 ± 4.3, 713.3 ± 26.7, and 586.3 ± 27.4 Ω) closely matched those averaged among all five mice (858.5 ± 113.5, 764.1 ± 103.6, and 702.6 ± 98.1 Ω). The largest measurement discrepancy between the device (1447 ± 19.5 Ω) and development kit (1008.9 ± 133.5 Ω) was between the N.T. tissue group, but similar values have been obtained in earlier experimental trials (Supplementary Fig. 13). It should be noted that the assembled device was somewhat sensitive to changes in capsule orientation, but this is to be expected due to the small size of mice tissue relative to the sensor. Ultimately, these promising results verify that the developed bioimpedance sensing capsule can characterize damage to intestinal mucosa, resulting in incremental reductions in bioimpedance magnitude.

In this work, we have demonstrated the viability of our bioimpedance sensor design via repeated characterization using porcine and mouse colonic tissue ex vivo, achieving an integrated device capable of wireless readout of depth-specific impedance of GI tissues toward in vivo diagnostics. Bioimpedance measurements performed on excised mice and porcine intestinal tissues confirm the device’s ability to characterize epithelial permeability with minimal measurement variation. Although the developed device isolates the impedance contributions of intestinal mucosa, the sensor design process presented in this work may be adapted for monitoring other types of epithelial tissues. Beyond IBD, altered tight junction integrity is implicated in other GI conditions^70^ such as diarrhea, Colitis, and IBS. Hence, the device presented in this work would broaden the array of diagnostic tools available for localized, continuous monitoring of leaky gut. Before transitioning to a clinical context, additional experimentation must first be conducted. For example, to accurately characterize mucosal barrier integrity in vivo, baseline bioimpedance values for various GI tissues must be established using the capsule, as they are known to vary between each region^71^. Integration of a pH sensor for localization would assist in differentiating impedance alterations attributed to tight junction dilation and capsule transition between GI regions during passage. For initial in vivo device evaluation, we aim to validate that the device can wirelessly report altered mucosal integrity induced through dextran sodium sulfate (DSS) challenge following implantation into a rat caecum. A study eventually employing the capsule in a porcine model should also be performed to assess how passage of bolus or luminal fluids affect impedance measurement. This experiment would also elucidate noise concerns caused by the harsh chemical environment of the GI tract, potentially altering PEDOT:PSS film conductivity and thus measured impedance^72^. Previous literature has demonstrated that PEDOT:PSS post-treatment in HCl- or HCOOH- enhances mechanical and chemical stability while mitigating sensitivity against H_2_O passage^73^. Additionally, a thorough investigation of the device’s packaging scheme is underway to confirm that luminal fluid does not penetrate the assembled capsule during transit. Finally, while this study evaluates bioimpedance as a diagnostic tool for assessing barrier integrity, we aim to eventually integrate the sensor with other capsule-based subsystems for feedback-driven intervention^74^. Ultimately, this work is a major step toward non-invasive characterization of intestinal mucosal integrity for real-time monitoring and diagnostics of the GI tract.

## Methods

### Microelectrode fabrication and PEDOT:PSS modification

Fabrication of the flexible bioimpedance sensor was achieved through a standard liftoff process onto 1 mil Kapton® polyimide film (DuPont, Wilmington, DE). First, the polyimide film was adhered to a 4”-silicon wafer by spin coating Omnicoat at 3000 rpm and adding a 16 µm layer of SU-8 2015 photoresist purchased from Kayaku Advanced Materials (Westborough, MA). Photomasks were designed using AutoCAD (Autodesk, San Rafael, CA). Electrode traces are patterned on a MLA150 Maskless Aligner (Heidelberg Instruments, Heidelberg, Germany) using Shipley S1813 photoresist (Kayaku Advanced Materials), followed by deposition of Cr/Au (20/70 nm) using electron-beam evaporation and subsequent lift-off with acetone for 15 min under sonication (Supplementary Fig. 2). The substrate was coated with Parylene C (1.6 µm) to insulate Au traces from the external environment using the Parylene Deposition System 2010 (Specialty Coating Systems, Johnstown, PA). The electrodes and contact pads were exposed by reactive ion-etching (RIE) with 100 SCCM O_2_ (at 100 mTorr) for 240 seconds at 50 W. Sensors were cleaned with acetone, methanol, and isopropanol (AMI); rinsed thoroughly with deionized water ( > 18.2 MΩ) from an E-pure Ultrapure Water Purification System (Thermo Scientific, Waltham, MA), and dried with N_2_. Lastly, sensors are cut into an L-shape to align the electrodes along the length of the capsule when connected to the capsule electronics. Next, the Au electrodes were modified via electrodeposition by immersing the sensor into a 200 µL working cell filled with 3–4% high conductivity grade poly(3,4-ethylenedioxythiophene) polystyrene sulfonate (PEDOT:PSS) (Sigma Aldrich, St. Louis, MO) and then performing chronopotentiometry at fixed current densities (1, 3, and 5 µA·mm^−2^) for 600 s using a BioLogic VSP300 potentiostat (Seyssinet-Pariset, France). A standard commercial Ag/AgCl reference and Pt wire counter electrode were purchased from CH Instruments (Bee Cave, TX) to complete the electrochemical working cell.

### Benchtop characterization of PEDOT:PSS-coated electrodes

EIS (*N* = 3) was performed in six KCl solutions ranging from 1 µM to 100 mM between 100 Hz and 200 kHz using the EVAL-AD5941 using the unmodified and PEDOT:PSS-coated bioimpedance sensor. The excitation signal used a peak-to-peak voltage of 100 mV_p-p_. EIS (*N* = 3) was performed in 1x PBS using an excitation signal of 100 mV_p-p_ at 15 min intervals for 150 min to evaluate time-dependent PEDOT:PSS impedance magnitude response.

### Capsule electronics and assembly

A custom flex-rigid printed circuit board (PCB) was designed as previously described^48^. The device uses a commercially available AFE (AD5941, Analog Devices) and low-power Bluetooth 5 MCU (BGM13S, Silicon Labs) to perform EIS measurements and wirelessly transmit data to a smart phone. A 2.45 GHz antenna (WLA.01, Taoglas) allows for wireless transmission (up to +18 dBm). Assembly and fabrication of the PCB was conducted by Sierra Circuits LLC (Sunnyvale, CA). The entire system is powered from a 3.0 V lithium manganese dioxide battery (2L76, Energizer), 10.6 mm in diameter, which is supplied through a 3.3 V voltage regulator (TPS610981, Texas Instruments). A magnetic reed switch (HSR-502RT, Hermetic Switch) was soldered directly to the PCB to disconnect the battery when not in use. The interrogation frequency of the system was fixed at 10 kHz based on prior sensor characterization. An impedance magnitude threshold was wirelessly set using the phone app to demonstrate the sensor feedback capabilities of the device. This threshold was determined to ‘turn on’ the onboard LED in response to a drop in magnitude below a threshold value indicative of increased tissue conductivity. The capsule electronics and battery are housed in an LCD 3D-printed capsule shell (Ø =13.8 mm), comprising FormLabs Surgical Guide v2 Resin. The flexible bioimpedance sensors are inserted into a four-pin flat-flex connector (FFC) and sensor port along the length of the capsule. Once inserted, the sensor is contoured around the exterior of the shell and sealed in place with hot-melt adhesive. A customized GATT profile was created containing fields for reading the fixed-frequency magnitude and phase through the EFR Connect app to sample impedance values using the BLE-MCU. Additionally, GATT fields for user data entry enables the impedance interrogation frequency and LED activation threshold to be dynamically set from the mobile device following capsule assembly.

### Ex vivo tissue characterization on porcine tissues

Porcine small intestinal tissue was procured from Animal Biotech Industries (Doylestown, PA) and stored at −20 °C until experimentation. One uniform small intestinal sample was further segmented into ten samples (1 cm^2^) and separated into test groups consisting of untreated (*N* = 5) and conductive tissues (*N* = 5) to compare measurement variability on intestinal tissue and establish baseline values for differentiating tissue while stationary. Samples were immersed in 1x phosphate-buffered saline (PBS) for 24 h and stored at 4 °C to alter tissue conductivity. Prior to testing, samples were acclimated to room temperature for 15 min. Unmodified and PEDOT:PSS-coated sensors were placed against the intestinal lumen with 5 g weight to normalize the contact pressure. EIS measurements were performed using an Analog Devices electrochemical evaluation PCB, EVAL-AD5941, and SensorPal GUI software to record the impedance magnitude and phase response. The excitation signal consisted of a 100 mV_p-p_ sinusoidal waveform with a frequency sweep ranging from 100 Hz to 200 kHz at 50 points per decade.

The impedance sensors were evaluated while moving across ex vivo tissue samples using a custom linear actuator to assess potential motion artifacts. Porcine small intestines were sectioned into two 5 cm segments and treated identically to the test groups in the stationary experiment. The PBS-soaked tissue was dyed blue for visualization purposes. Each sample was opened luminally and pinned to a silicone tissue holder with a 25 mm intrusion, mimicking the diameter of human intestinal lumen. The linear actuator for capsule translation consisted of a stepper motor with lead screw and an Arduino microcontroller which controlled the relative position, speed, and acceleration of the capsule along the lead screw track. A 3D-printed polylactic acid (PLA) weight holder was created, allowing the capsule to maintain consistent contact pressure under gravity (Supplementary Fig. 9). The bioimpedance-sensor integrated capsule was translated at a rate of 3 mm·s^−1^ mimicking peristaltic motion. Impedance magnitude was wirelessly reported to a mobile device via the EFR Connect app (Silicon Labs, Austin, TX).

### EDTA challenge on mice colonic tissues

Mouse colonic tissue samples were extracted from seven C57BL/6 mice, opened luminally, and washed in 1x PBS solution. All samples were preserved in Kreb’s-Ringer bicarbonate buffer (KRB) with carbogen gas (95% O_2_, 5% CO_2_) purchased from Airgas (Radnor, PA). Samples were exposed to increasing concentrations of EDTA, a calcium chelator that disrupts adherens and tight junctions, to alter tissue permeability at varying rates of reaction. For measuring TEER, excised tissue from one mouse was divided into four samples and placed into an Ussing chamber (P2300, Physiologic Instruments). Of these samples, two are treated with EDTA in concentrations of 1 mM, 3 mM, and 5 mM at 100 s, 1400 s, and 1800 s, respectively, and two were used as a non-treated control. Conductivity was recorded for 2600 s.

For characterizing the fabricated bioimpedance sensors, excised tissue from the remaining six mice (Mouse I-VI) were segmented into ~1 cm^2^ samples and pinned to a foam biopsy sponge. Before each measurement, samples are stabilized in an oxygenated KRB solution for 3 min, treated in a mixture of KRB solution and varied EDTA concentrations (0, 1, 3, 5, 10 mM) for 1 min, followed by a 30 s rinse in KRB as shown in Fig. 4a. Samples are returned to the original oxygenated KRB solution for measurement. Control measurements are performed on non-treated (N.T.) tissue samples and the KRB-soaked sponge to normalize the data across multiple mice. EIS was performed with an excitation signal of 600 mV_p-p_ at interrogation frequencies ranging from 100 Hz–200 kHz. Mouse I-V were measured using the Analog Devices electrochemical evaluation PCB, EVAL-AD5941, and Mouse VI was measured using the wireless ingestible capsule.

### Statistics and reproducibility

For full-frequency bioimpedance measurements performed before and after PEDOT:PSS coating, as well as bioimpedance measurement on excised porcine and mice tissue, standard deviation (SD) was used to evaluate the reproducibility of the devices. A sample size of *N* = 3 or more was used for all measurements with exception to translation across porcine tissue and full-frequency wired mice experiments. During mice testing, for each tissue group, *N* = 4 full-frequency measurements are performed per EDTA treatment group, and tissue samples are returned to oxygenated KRB solution between each measurement for preservation. No statistical method was used to predetermine sample size. The investigators were not blinded to allocation during experiments and outcome assessment. An unpaired T-test was employed on the *N* = 4 unmodified impedance magnitude values sampled at 10.5 kHz to assess differences in average bioimpedance magnitude between treatment groups on individual mouse samples, with results shown in Fig. 4c. Next, a paired T-test was employed to assess averaged magnitude differences for all five mice. The *T*-test (*N* = 5) used paired average magnitude for each sample under the various treatment conditions (Fig. 4d) to ensure that natural variations in mice tissue thickness and sensors did not bias the results. In accordance with standard scientific practices, the baseline significance threshold for determining statistical relevance was set at *P* = 0.05.

## Supplementary information

Device placement on unmodified and phosphate-buffered saline soaked porcine intestinal tissue

Device translation across porcine intestinal tissue during wireless measurement

Supplemental Information

Dataset 1

## Data Availability

All data generated that supports the findings of this study are available in the source data file provided with the manuscript.
