# Supplementary material for: An ingestible bioimpedance sensing device for wireless monitoring of epithelial barriers
Source: Microsyst Nanoeng. 2025 Feb 7;11:24. doi: 10.1038/s41378-025-00877-8 (PMC11802857; doi:10.1038/s41378-025-00877-8)
Supplement: Supplementary file 3 — Supplemental Information [file 41378_2025_877_MOESM3_ESM.pdf]

An ingestible bioimpedance sensing device for wireless monitoring of epithelial barriers

Brian M. Holt[1,2], Justin M. Stine[2], Luke A. Beardslee[2], Hammed Ayansola[4], Younggeon Jin[4], Pankaj J. Pasricha[5], and Reza Ghodssi[1,2,3*]

[1]Deparment of Electrical and Computer Engineering, University of Maryland, College Park, MD, 20740, USA.

[2]Institute for Systems Research, University of Maryland, College Park, MD, 20740, USA.

[3]Robert E. Fischell Institue for Biomedical Devices, University of Maryland, College Park, MD, 20742, USA.

[4]Deparment of Animal & Avian Sciences, University of Maryland, College Park, MD, 20740, USA.

[5]Mayo Clinic Hospital, Pheonix, AZ, 85054, USA.

E-mail: ghodssi@umd.edu

Table of Contents

Supplementary Table 1. Capsule component list and power consumption……………………..………… 3

Supplementary Table 2. Ingestible device size comparison…………………...……………………….... 4

Supplementary Fig. 1. Observed interrogation frequency limits with AD5941…………………………… 5

Supplementary Fig. 2. FEM modeling of electrode geometries…………………………………………… 6

Supplementary Fig. 3. Fabrication process flow…..…………..…………………………………………7

Supplementary Fig. 4. Benchtop validation of bare Au depth-targeting on agar…………………..………. 8

Supplementary Fig. 5. PEDOT:PSS electropolymerization setup illustration ...…….……………………. 9

Supplementary Fig. 6. Chronopotentiometry recording without replacing working cell...…….…………. 9

Supplementary Fig. 7. Cyclic voltammetry comparison of various deposition times..…….……………….. 10

Supplementary Fig. 8. Stationary measurement with untreated sensor integrated capsule...…….……… 11

Supplementary Fig. 9. Linear actuator for capsule translation across tissue …………………………….. 12

Supplementary Fig. 10. Initial impedance measurement on tissue following integration with capsule ….12

Supplementary Fig. 11. Full-frequency impedance magnitude using EVAL-AD5941 on mice colon ….. 13

Supplementary Fig. 12. Verification of negligible EDTA effect on measured impedance ………….…... 14

Supplementary Fig. 13. Early experimentation with five-minute EDTA exposure …………...…………15

Supplementary References……………………………………………………………………………...16

Ingestible Capsule	Manufacturer Part #	Dimension (mm)	Description
**Analog Front End**	AD5941<br>Analog Devices	7.0 x 7.0 x 1.0	Idle: 0.56 mA<br>Amperometry: 4.0 mA (w/ ADC)<br>Spectroscopy: 9.1 mA (w/ ADC)<br>50 kHz: 1.6 mA (w/ ADC)
**Microcontroller**	BGM13S<br>Silabs	6.5 x 6.5 x 1.0	Radio Active: 9.7 mA<br>Low-power: 2.6 µA
**2.4 GHz Antenna**	WLA.01<br>Taoglas	3.2 x 1.6 x 0.5	Peak Gain: +2.5 dBi
**3V Coin Cell**	2L76<br>Energizer	Dia: 11.6; H: 10.6	Capacity: 160 mAh<br>Chem: Lithium/Manganese Dioxide
**Voltage Regulator**	TPS610981<br>Texas Instruments	1.5 x 1.5 x 0.75	3.3 V output<br>High Power mode: 15 µA<br>Low Power mode: 300 nA
**Analog Switch**	DG2735ADN<br>Texas Instruments	1.8 x 1.4 x 0.55	Supply Current: 1 µA
**Memory (FRAM)**	MB85RS64TPN<br>Fujitsu	2.0 x 3.0 x 0.75	Standby mode: 9 µA<br>Operational mode: 0.8 mA

Supplementary Table 1. Ingestible device component list, featuring dimensions and energy consumption. Under standard operating conditions, the capsule device can be continuously powered for up to 29 hours.

Reference	Capsule Size (Length x Diameter)	Number of Pigs	Pig Size (kg)
[1]	"000"	6	25
[2]	"00"	1	42
[3]	26 mm x 13 mm	4	17
[4]	40 mm x 10 mm	1	50
[5]	27 mm x 12 mm	6	40
[6]	37.5 mm x 15 mm	1	(?)
[7]	27 mm x 11 mm	14	50
[8]	12.5 mm x 10 mm (before deployment)	6	65
[9]	(?) x 10.5 mm	1	60
[10]	"000"	5	75
Developed	27.9 mm x 13.8 mm		

Supplementary Table 2. Comparison of developed device size with previously developed ingestible capsule devices evaluated in porcine animal models.

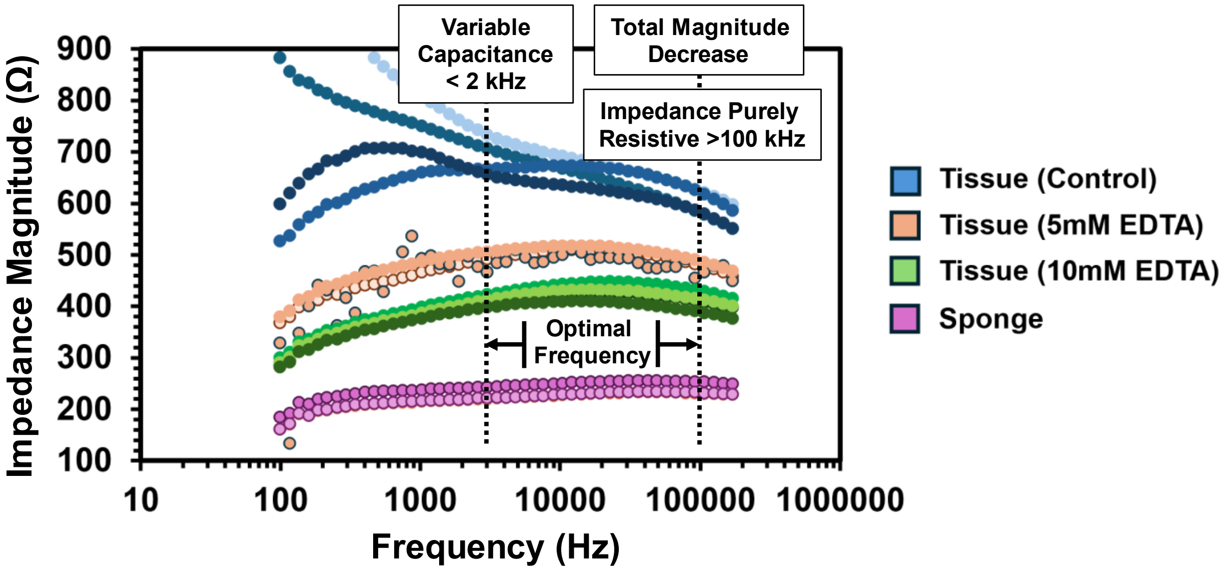

Supplementary Fig. 1. Electrochemical impedance spectroscopy (EIS) magnitude recording (N=4) between 100Hz and 170kHz on one sample of excised mouse colonic tissue. To enhance transepithelial permeability, tissue is treated in 5mM and 10mM ethylenediaminetetraacetic acid (EDTA) for three- and five-minute intervals, respectively. The tissue is preserved in Kreb's Ringer Bicarbonate Buffer (KRB) and pinned to a soaked foam biopsy sponge located beneath the tissue. Measurements are collected via PC using the EVAL-AD5941 development kit with the fabricated sensor following treatment with poly(3,4-ethylenedioxythiophene) polystyrene (PEDOT:PSS). Colored in various shades of pink are baseline impedance magnitude values of the KRB-soaked sponge, along with untreated (blue) and treated tissue (orange, green). Below 2 kHz, variation in measured magnitude is quite high due to dominant capacitances. Above 100 kHz, capacitive effects become largely negligible, resulting in a decrease in measured magnitude. Between 2 kHz and 100 kHz, measured impedance is stable and optimal for evaluation of tight junction integrity.

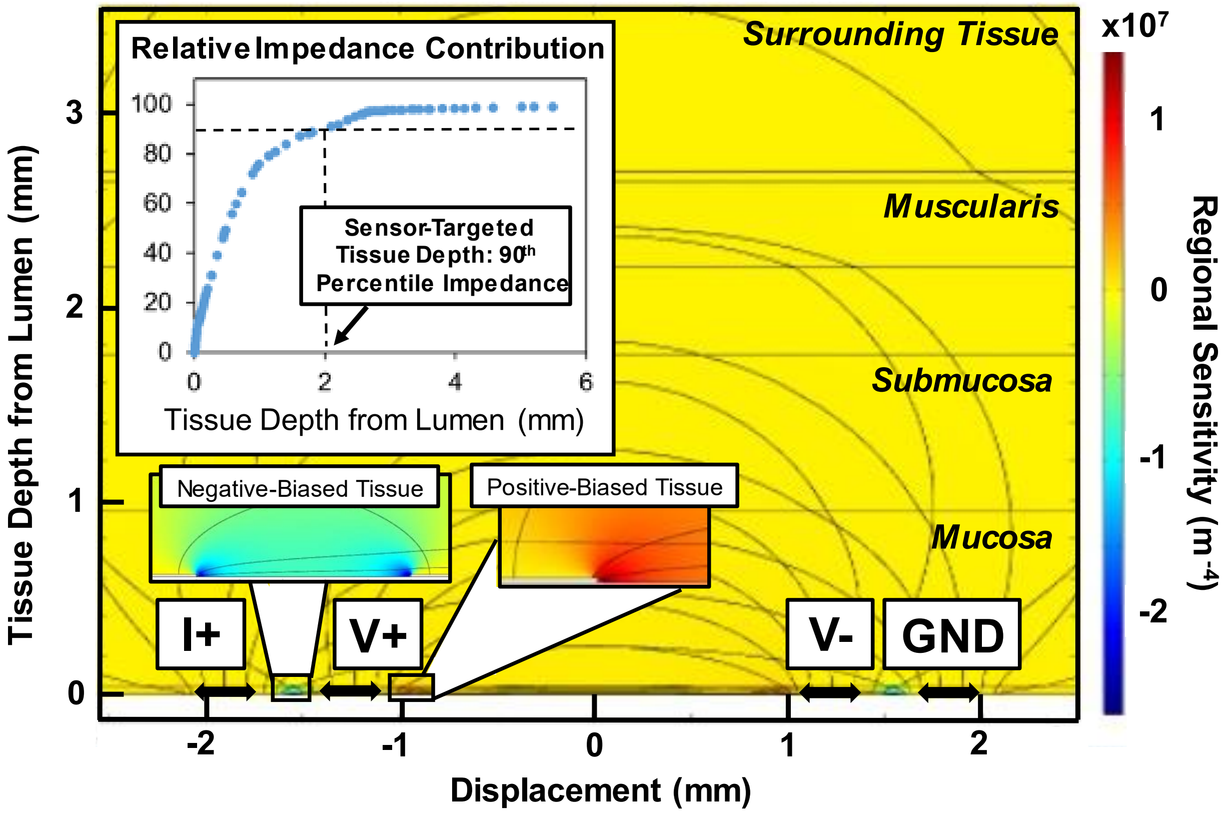

Supplementary Fig. 2. Finite-element model devised in COMSOL featuring tetrapolar gold electrode array interfacing with stacked intestinal tissue layers with dielectric properties obtained from P. Kassanos et al.[11] Electric field lines propagate from each electrode through each intestinal tissue layer and positive-sensitive tissue regions are highlighted in red, whereas negative-sensitive regions are highlighted in blue. The featured geometry uses an electrode width of 500 µm and inner distance of 2 mm. By taking the volume integral of the sensitivity field with respect to distance from the electrodes, it was found that about 90% of measured impedance prioritizes tissue located with a depth of 2 mm from the lumen (inset). To further prioritize the mucosal layer, an inner distance of 1 mm was chosen for the final design.

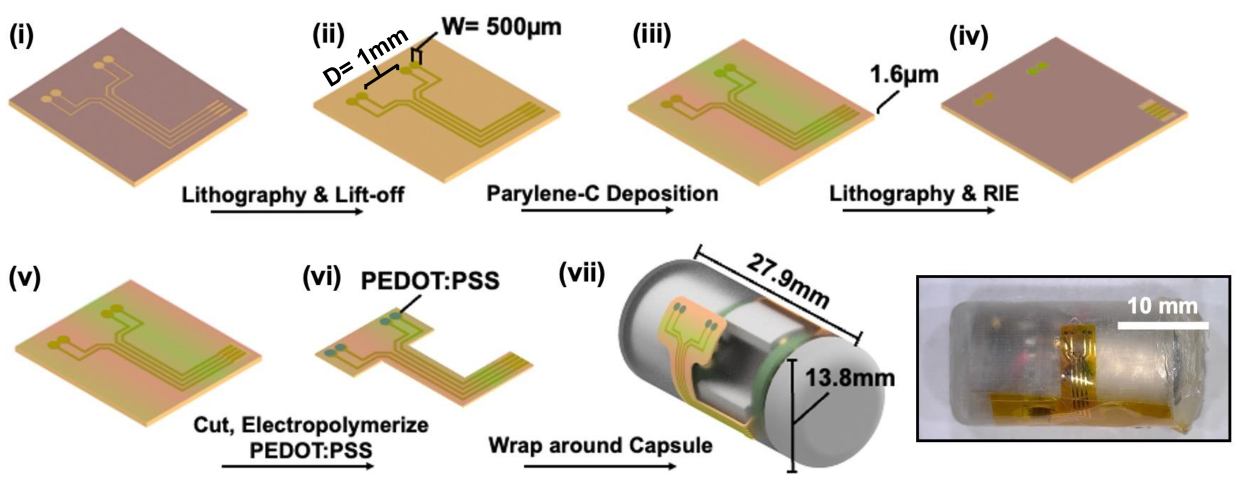

Supplementary Fig. 3. Summary of bioimpedance sensor fabrication process and capsule prototype assembly. (i-iii) Lift-off process results in patterned Cr/Au traces on Kapton film. Sensors are cut and bent to align along the length of the capsule. (iv-vi) Note that CAD renderings are not drawn to scale, but inner electrode spacing is exaggerated for visualization. The assembled electronics are inserted into the 3D-printed shell exposing a port for the impedance sensor, which is sealed using biocompatible epoxy. Final capsule dimensions are 13.75 mm in diameter and 27.9 mm in length.

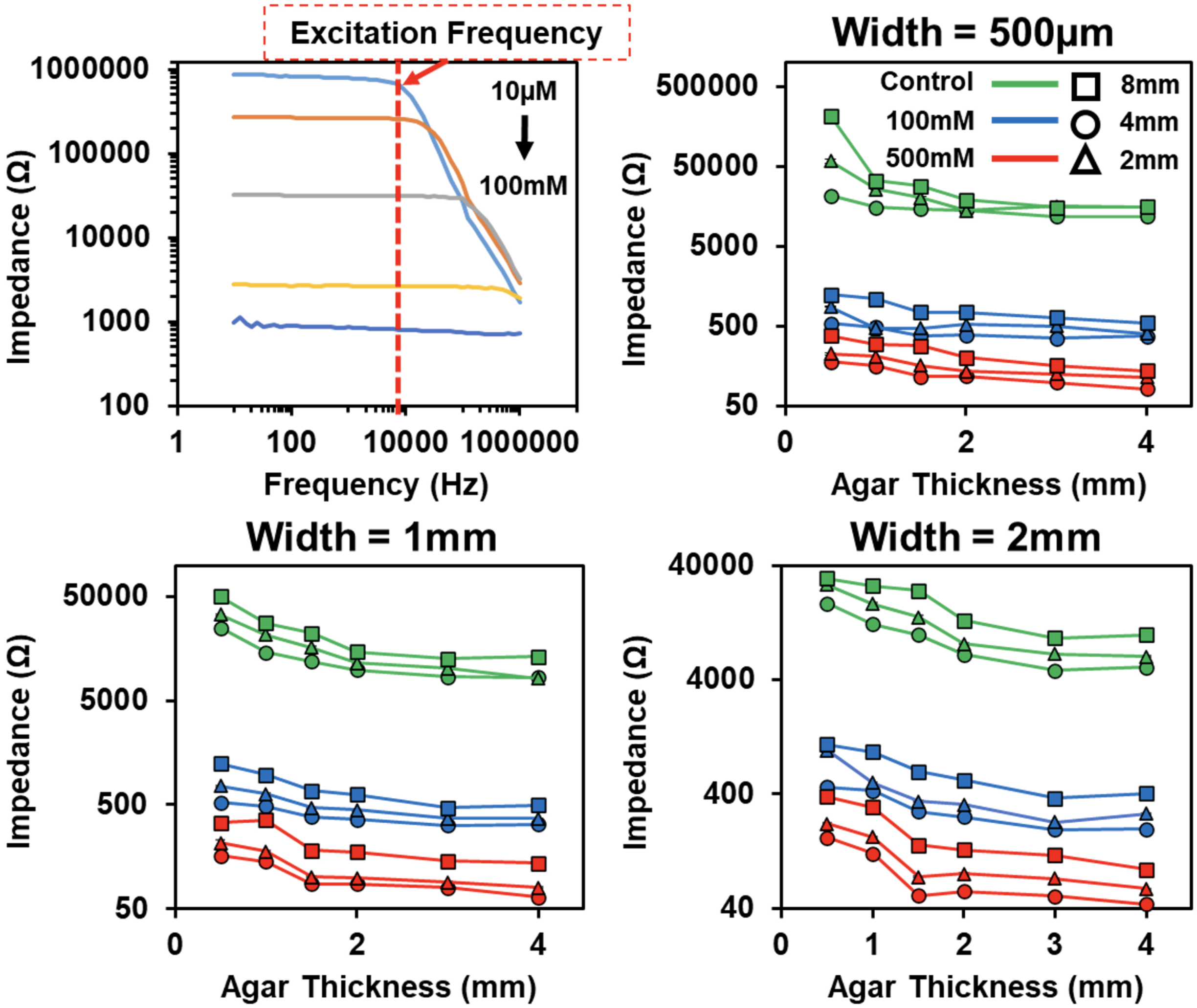

Supplementary Fig. 4. Characterization of bare Au tetrapolar bioimpedance sensors without PEDOT:PSS treatment using a benchtop Gamry Interface 1010E potentiostat. Various sensor geometries were fabricated and impedance was measured on agarose tissue phantoms of various thickness housed in a dielectric 3D-printed tub. This experiment was performed to visualize the impedance plateau behavior observed in the FEM model in response to electrode geometry to selection a geometry which isolates mucosal tissue impedance contributions. a) Full-spectrum impedance spectroscopy using electrode width W=500µm and inner distance D=4mm in 100mM NaCl aqueous solution. (b-d) Impedance recordings (at 10kHz) of electrodes of diameter (500µm, 1mm, and 2mm) and spacing (2mm, 4mm, and 8mm) on agar phantoms of three NaCl concentrations (0mM, 100mM, and 500mM), estimating target depth of the sensor .

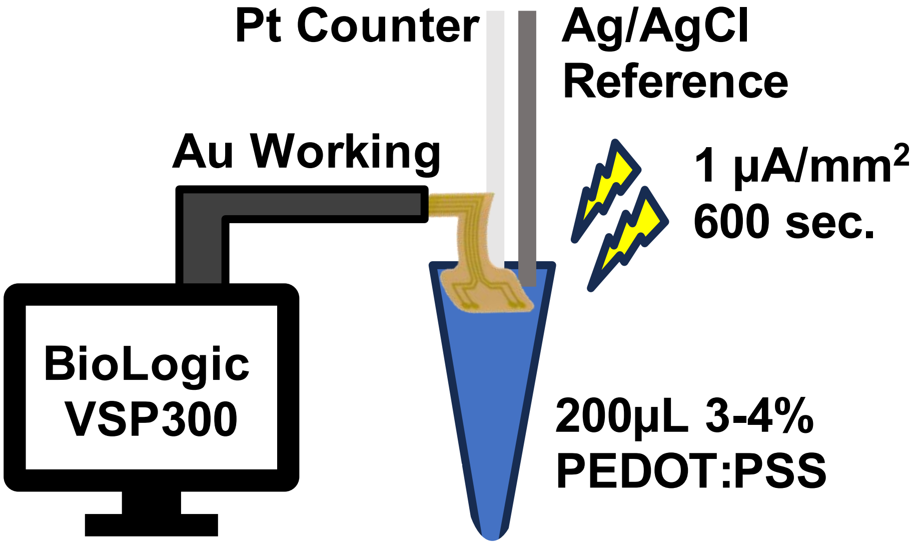

Supplementary Fig. 5. Electrode setup for PEDOT:PSS electropolymerization using a standard Ag/AgCl reference electrode and Pt counter electrode. The working cell contained 200 μL of 3-4% high conductivity grade PEDOT:PSS (Sigma Aldrich, St. Louis, MO, USA) and galvanostatic deposition was used for 600 seconds.

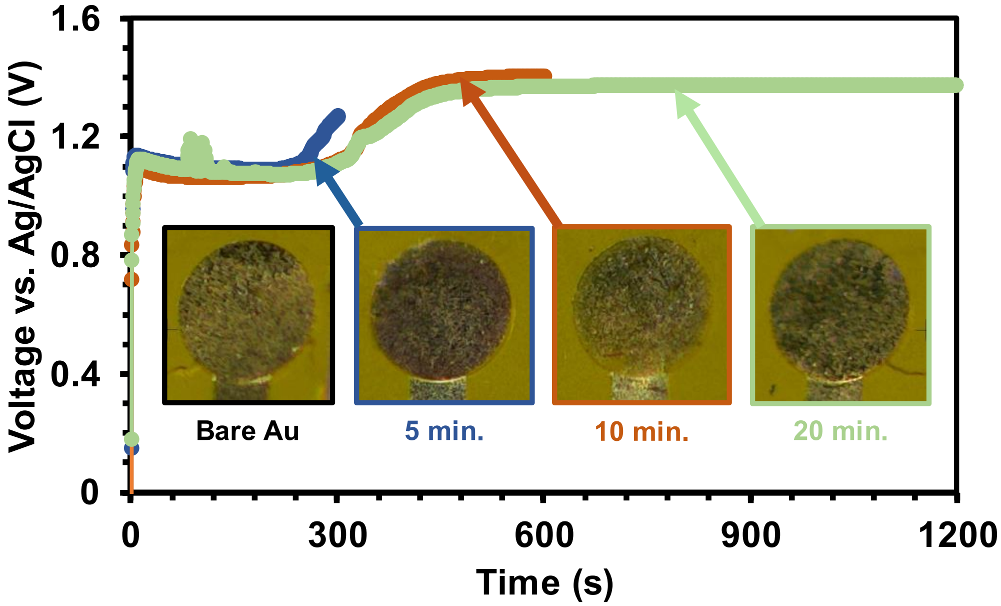

Supplementary Fig. 6. Chronopotentiometry recording during single-electrode PEDOT:PSS coating without changing the working cell using a galvanostatic current density of 1μA/mm^2. Note that the initial plateau in working electrode voltage occurs earlier for the initial 5 min. treatment, indicating that subsequent electropolymerization processes take longer to complete. Additionally, discoloration is most evident during initial coating, and less visible during subsequent coatings.

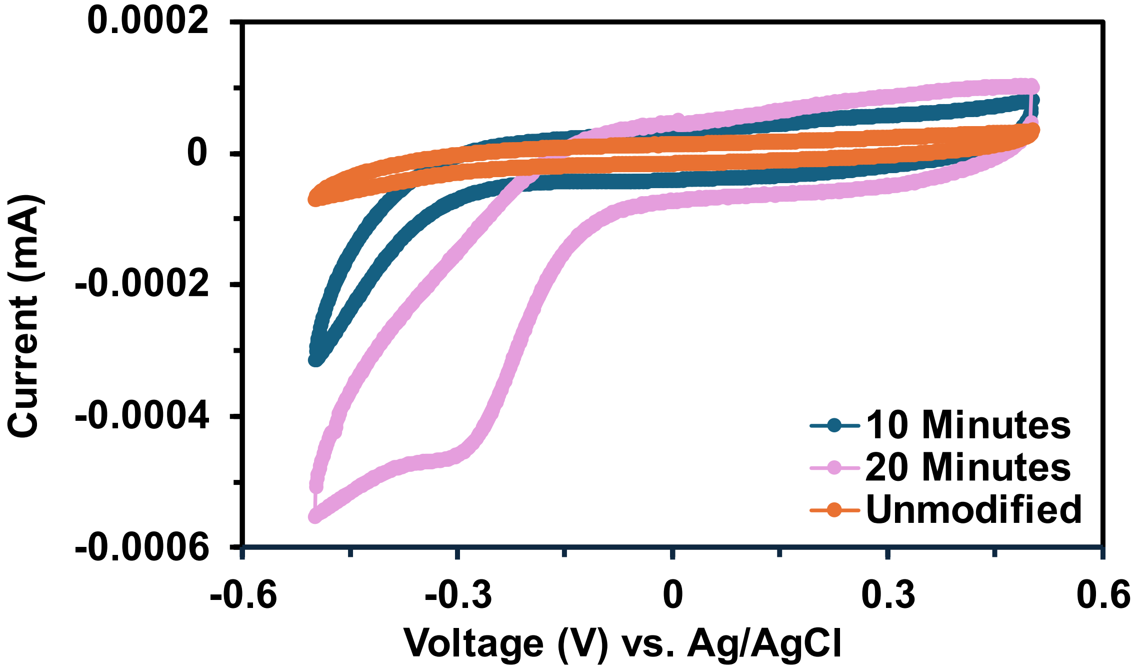

Supplementary Fig. 7. Cyclic voltammetry in 1x phosphate-buffered saline confirms that subsequent treatments without changing the working cell offer little to no enhancement in charge transfer capability. Hence, in the final sensor treatment, all four electrodes are simultaneously coated using the same working cell to minimize variability in charge transfer capability between electrodes. The largest impact on charge transfer density was the amount of PEDOT:PSS in the working cell as well as initial deposition current density, since subsequent coatings offered limited improvement in interfacial impedance resolution. Thus, a deposition time of 600 seconds using the low current density 1μA/mm^2 using fresh working cells for each sensor was found to be optimal.

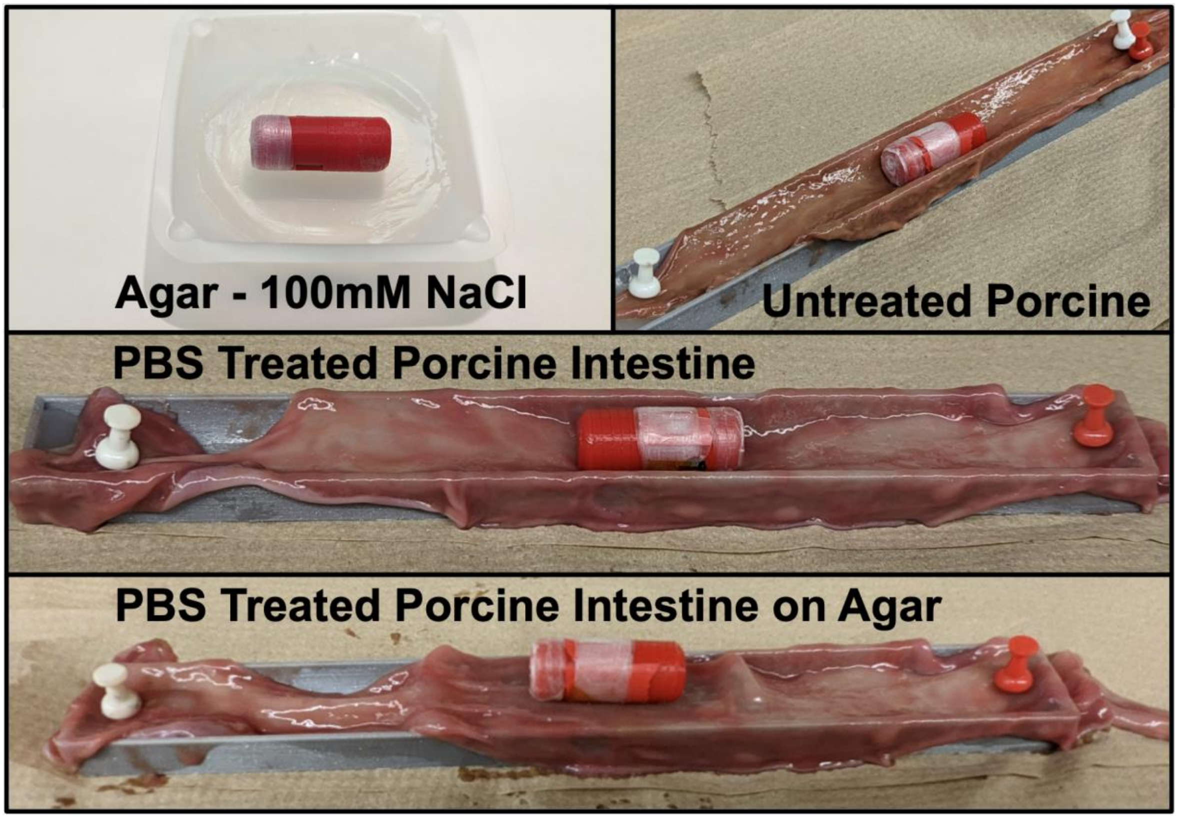

Supplementary Fig. 8. Evaluation of packaged bare Au bioimpedance sensor on (a) an agar phantom (100mM NaCl), (b) untreated and (c) treated porcine small intestinal tissue ex-vivo, and (d) placed on agar backing to simulate a "conductive media," such as surrounding tissues. Capsule electronics including the AD5941 were housed in a 3D-printed polylactic acid shell prototype package and the impedance excitation signal used was 10kHz frequency at 50mVp-p. Mesentery is removed from the porcine tissue prior to experimentation for the agar backing to be used directly beneath the tissue. The geometry featured in this experiment is an electrode width of W= 500 µm and inner distance of D= 1 mm. As expected, the sensor integrated capsule exhibited a decrease in impedance on PBS-soaked porcine tissue (3547 ± 11 Ω) compared to untreated tissue (4781 ± 154 Ω). The tissue was subsequently placed on the agarose tissue phantom (5461 ± 24 Ω) to assess the degree to which impedance is further reduced. If the impedance measurement is significantly affected, then the sensor geometry is not targeted toward the epithelial layer of the porcine tissue. Minimal impedance change was observed following tissue placement on the agar phantom (3438 ± 9 Ω), confirming a limited target depth. Though stationary measurement confirmed the depth-targeted nature of the fabricated sensor, observed impedance magnitude was quite high on PBS-treated tissue and very sensitive to capsule placement and orientation, warranting the initial investigation into the reduction of interfacial impedances.

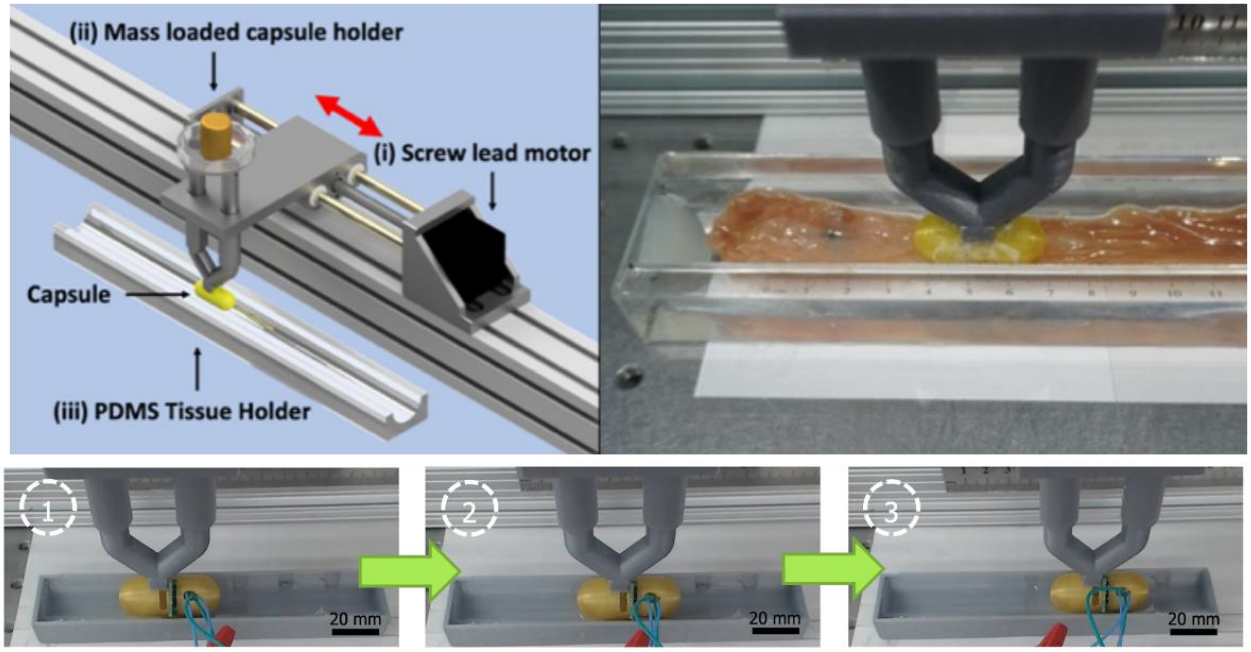

Supplementary Fig. 9. A linear actuator was constructed consisting of a stepper motor connected to an Arduino microcontroller. A 3D-printed polylactic acid (PLA) attachment may be inserted into the body of the holder so that a weight holder can allow the device to maintain consistent contact pressure under gravity.

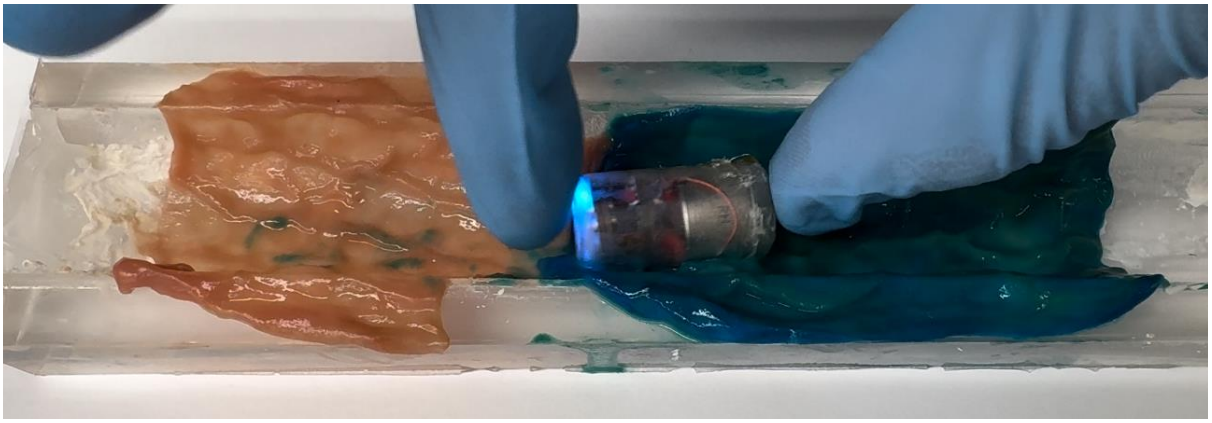

Supplementary Fig. 10. Initial placement of capsule on non-treated (N.T.) and phosphate-buffered saline (PBS) soaked dyed porcine small intestinal tissue model. Reported impedance values used to wirelessly set a magnitude threshold via the EFR Connect application on mobile device to verify capsule ability to differentiate tissue. As pictured, capsule LED activates upon measured impedance magnitude of 650Ω or less.

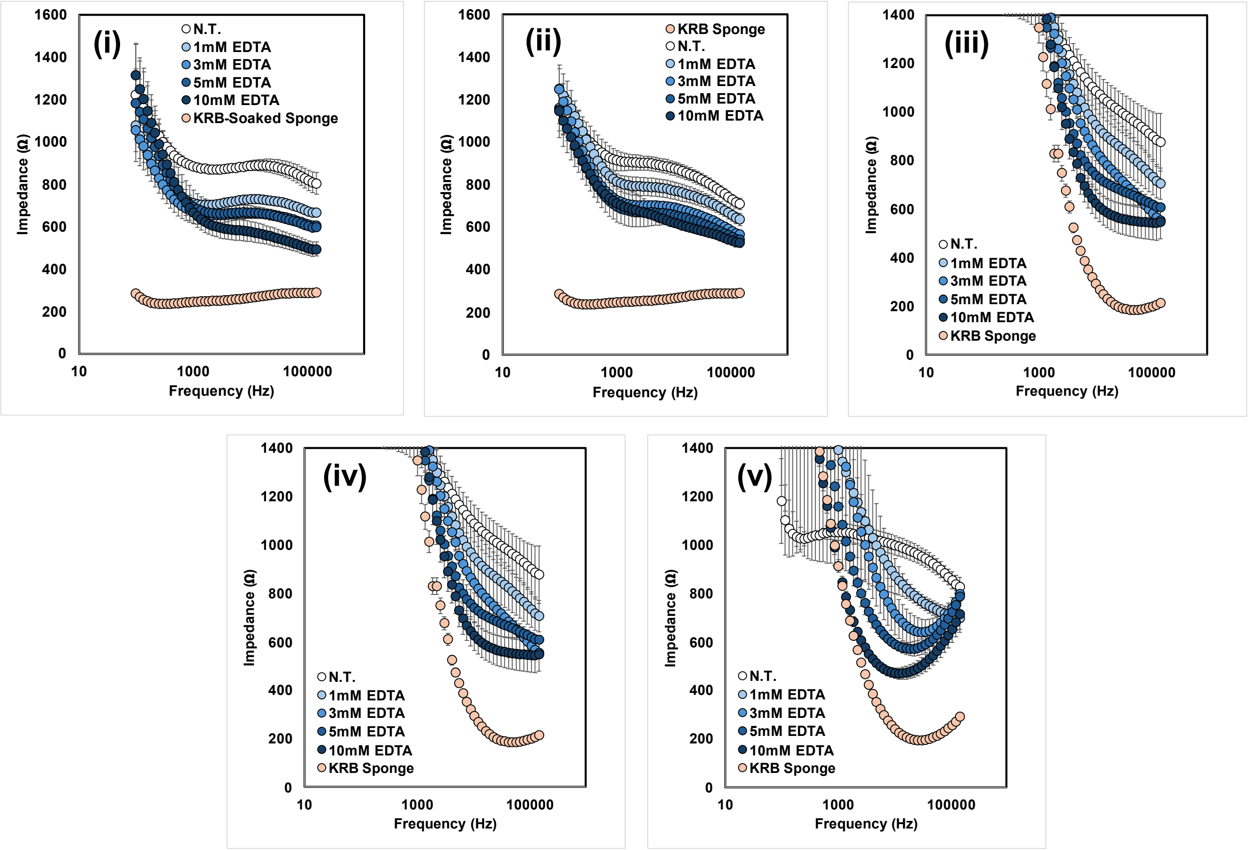

Supplementary Fig. 11. Mice I-V full-frequency impedance magnitude response between 100kHz and 170kHz before and after treatment of 1mM, 3mM, 5mM, and 10mM EDTA. Measurements are collected using the fabricated sensor connected to the EVAL-AD5941 development kit. Colored in orange is the baseline magnitude response of the KRB-soaked sponge located beneath the tissue.

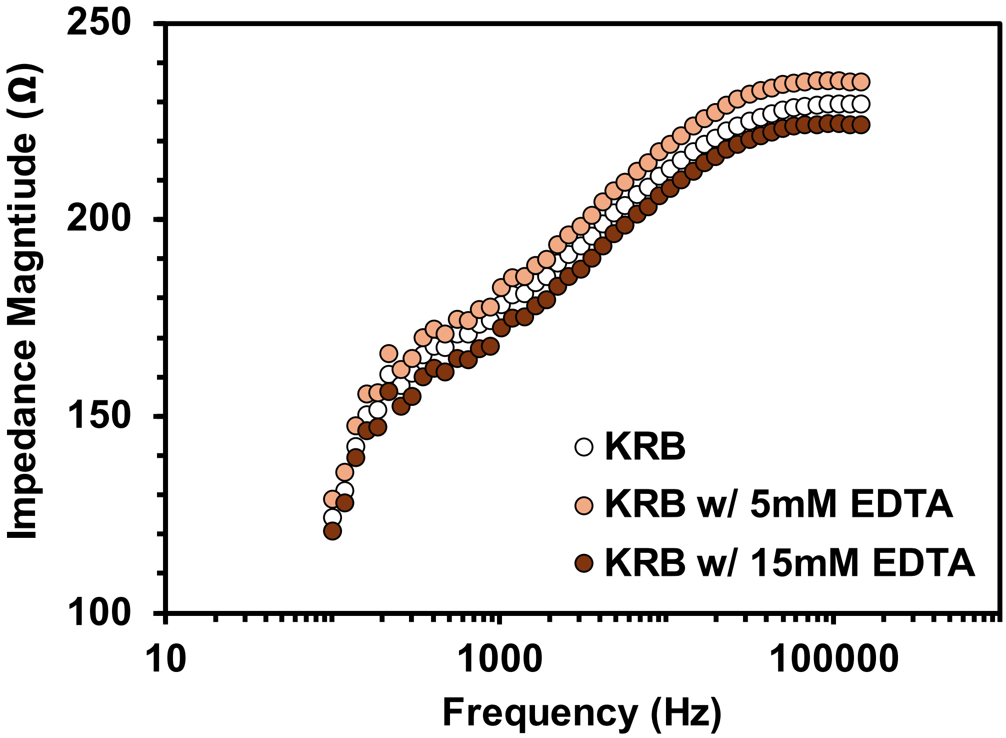

Supplementary Fig. 12. Wired impedance magnitude measurement obtained using fabricated PEDOT:PSS treated sensor with EVAL-AD5941 in Kreb's-Ringer Bicarbonate (KRB) glucose solution with increasing EDTA concentration. It is evident that EDTA itself has negligible effect on measured impedance magnitude.

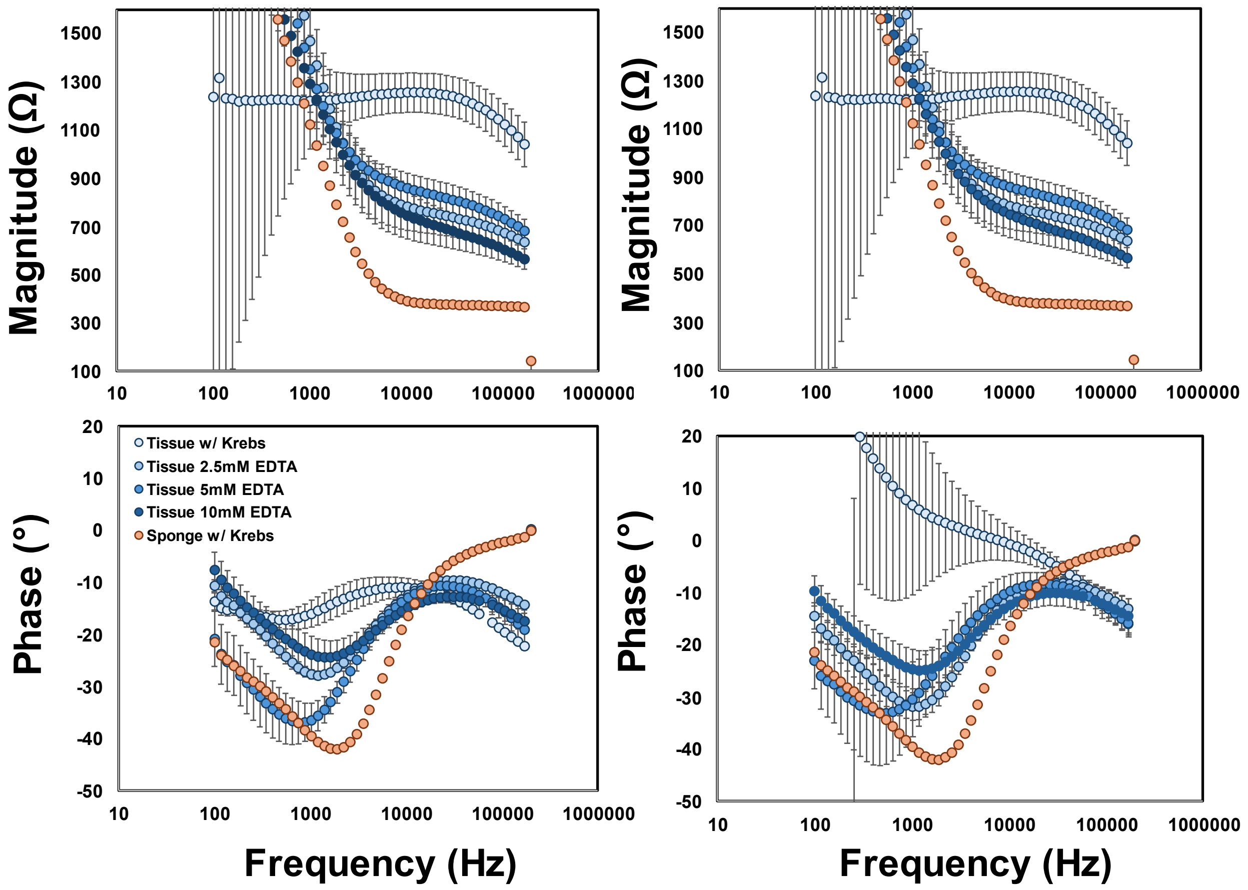

Supplementary Fig. 13. Wired impedance magnitude and phase measurement obtained using fabricated PEDOT:PSS treated sensor with EVAL-AD5941 on mouse colonic tissue. Unlike the one-minute treatment intervals performed in the five-mice trial, two samples of tissue were exposed to EDTA concentrations of 2.5 mM, 5 mM, and 10 mM for five minutes each. Although baseline impedance measurement on healthy colonic tissue aligned with results obtained using the assembled capsule device, the extended treatment duration resulted in little magnitude difference between EDTA-treated groups, suggesting saturated mucosal permeability. Additionally, no clear relationship was observed between phase and induced tight junction dilation

Supplemental References

1. Sarker, S. *et al.* A Novel Capsule-Delivered Enteric Drug-Injection Device for Delivery of Systemic Biologics: A Pilot Study in a Porcine Model. *IEEE Trans Biomed Eng* **69**, 1870–1879 (2022).
2. Chen, K. *et al.* An Edible and Nutritive Zinc-Ion Micro-supercapacitor in the Stomach with Ultrahigh Energy Density. *ACS Nano* **16**, 15261–15272 (2022).
3. Henze, L. J. *et al.* Characterization of gastrointestinal transit and luminal conditions in pigs using a telemetric motility capsule. *European Journal of Pharmaceutical Sciences* **156**, 105627 (2021).
4. Lee, J. H., Traverso, G., Ibarra-Zarate, D., Boning, D. S. & Anthony, B. W. Ex Vivo and In Vivo Imaging Study of Ultrasound Capsule Endoscopy. *J Med Device* **14**, (2020).
5. Orsini, B. *et al.* Ingestible light source for intragastric antibacterial phototherapy: a device safety study on a minipig model. *Photochemical & Photobiological Sciences* **22**, 535–547 (2022).
6. Xie, W. Design and validation of an in vivo long-term attachment capsule robot. (University of Nebraska, Lincoln, NE, 2017).
7. Schuster, K. F., Thompson, C. C. & Ryou, M. Preclinical study of a novel ingestible bleeding sensor for upper gastrointestinal bleeding. *Clin Endosc* **57**, 73–81 (2024).
8. Abramson, A. *et al.* An ingestible self-orienting system for oral delivery of macromolecules. *Science (1979)* **363**, 611–615 (2019).
9. Sang Heun Lee *et al.* A Wideband Spiral Antenna for Ingestible Capsule Endoscope Systems: Experimental Results in a Human Phantom and a Pig. *IEEE Trans Biomed Eng* **58**, 1734–1741 (2011).
10. Abid, A. *et al.* Wireless Power Transfer to Millimeter-Sized Gastrointestinal Electronics Validated in a Swine Model. *Sci Rep* **7**, 46745 (2017).
11. Kassanos, P., Ip, H. M. D. & Yang, G.-Z. A tetrapolar bio-impedance sensing system for gastrointestinal tract monitoring. in *2015 IEEE 12th International Conference on Wearable and Implantable Body Sensor Networks (BSN)* 1–6 (IEEE, 2015). doi:10.1109/BSN.2015.7299403.
